# Supplementary material for: CryoET reveals organelle phenotypes in huntington disease patient iPSC-derived and mouse primary neurons
Source: Nat Commun. 2023 Feb 8;14:692. doi: 10.1038/s41467-023-36096-w (PMC9908936; doi:10.1038/s41467-023-36096-w)
Supplement: Supplementary file 1 — Supplementary Information [file 41467_2023_36096_MOESM1_ESM.pdf]

SUPPLEMENTARY INFORMATION

CryoET Reveals Organelle Phenotypes in Huntington Disease Patient iPSC-Derived and Mouse Primary Neurons

Gong-Her Wu[1,*], Charlene Smith-Geater[2,*], Jesús G. Galaz-Montoya[1], Yingli Gu[3], Sanket R. Gupte[4], Ranen Aviner[5], Patrick G. Mitchell[6], Joy Hsu[4], Ricardo Miramontes[7], Keona Q. Wang[8], Nicolette R. Geller[8], Cathy Hou[1], Cristina Danita[1], Lydia-Marie Joubert[6], Michael F. Schmid[6], Serena Yeung[4,9], Judith Frydman[5,10], William Mobley[3], Chengbiao Wu[3], Leslie M. Thompson[2,7,8,11,12,+], Wah Chiu[1,6,13+]

[1]Department of Bioengineering, James H. Clark Center, Stanford University, Stanford, CA 94305, USA.

[2]Department of Psychiatry & Human Behavior University of California Irvine, Irvine, CA 92697, USA.

[3]Department of Neurosciences, University of California San Diego, La Jolla, CA, 92037-0662 USA.

[4]Department of Computer Science, Stanford University, Stanford, CA 94305, USA.

[5]Department of Biology, Stanford University, Stanford, CA 94305, USA.

[6]Division of CryoEM and Bioimaging, SSRL, SLAC National Accelerator Laboratory, Stanford University, Menlo Park, CA 94025, USA.

[7]Department of Memory Impairment and Neurological Disorders, University of California Irvine, Irvine, CA 92697, USA.

[8]Department of Neurobiology and Behavior, University of California Irvine, Irvine, CA 96267, USA.

[9]Department of Biomedical Data Science, Stanford University, Stanford, CA 94305, USA.

[10]Department of Genetics, Stanford University, Stanford, CA 94305, USA.

[11]Sue & Bill Gross Stem Cell Research Center, University of California Irvine, Irvine, CA 96267, USA.

[12]Department of Biological Chemistry, University of California Irvine, Irvine, CA 92617, USA.

[13]Department of Microbiology and Immunology, Stanford University, Stanford, CA 94305, USA.

* These authors contributed equally

+ These authors jointly supervised the work

To whom correspondence should be addressed:

Leslie M. Thompson; lmthomps@uci.edu; Wah Chiu; wahc@stanford.edu

SUPPLEMENTARY FIGURES AND TABLES

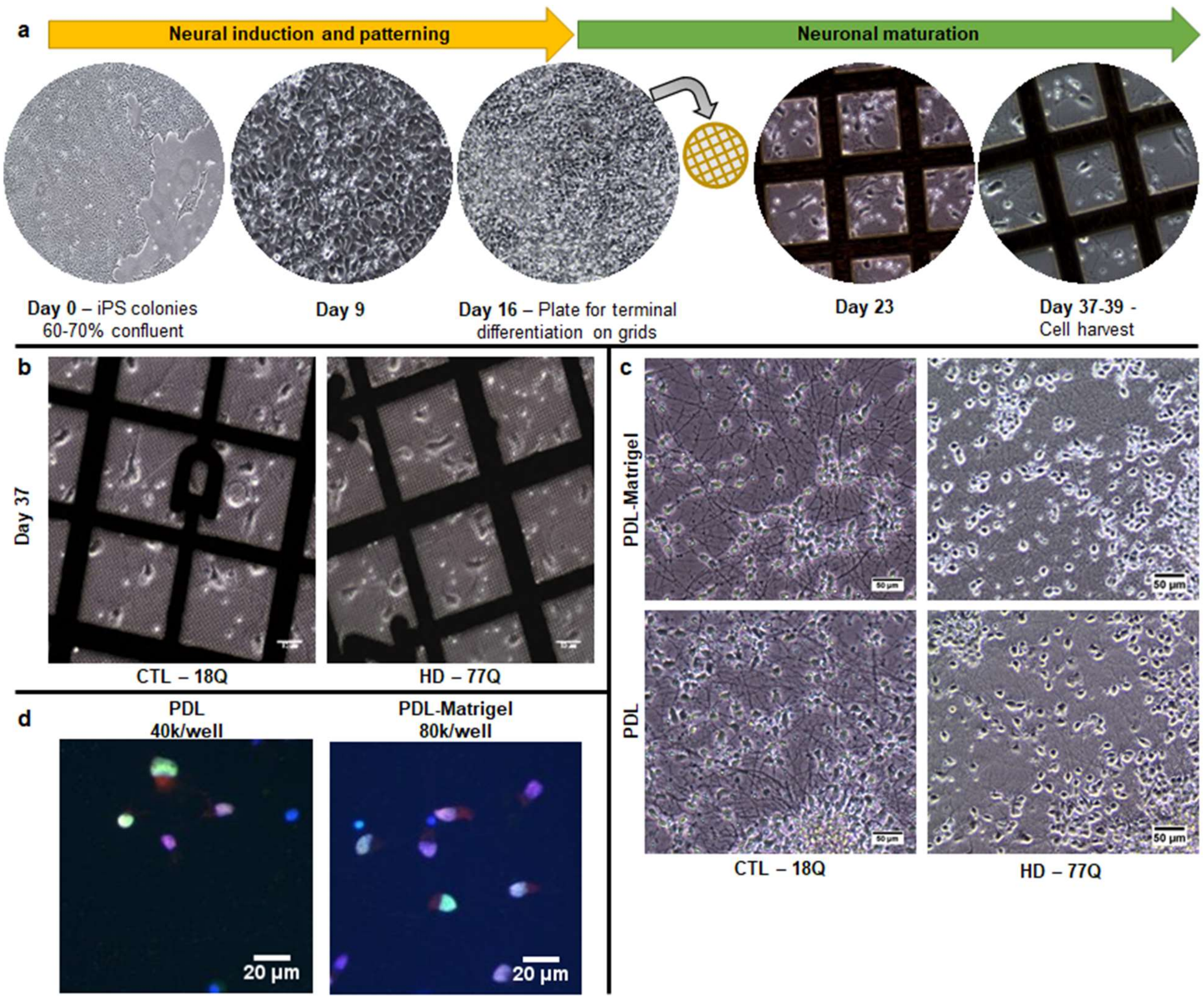

Supplementary Figure 1

Supplementary Fig. 1. Differentiation of control and HD iPSC-derived neurons for cryoET. a Differentiation paradigm outlining our previously published general protocol[1], introducing here the modification of plating cells at day 16 onto carbon grids for cryoET. **b** Representative phase-contrast images of day 37 neurons that were grown on grids coated with PDL alone, adapted from published protocol. Scale bar = 15 µm. **c** Representative images of control and HD Day 37 iPSC-derived neurons differentiated as previously described on PDL and Matrigel and compared to just PDL alone. Scale bar = 50 µm. This experiment has been performed twice using 2 and 6 cell lines. **d** Immunofluorescence of the 53Q line for two MSN markers, CTIP2 in green and DARPP32 in red, at our experimental setup for the grids on the left and cultured as previously described on the right, experiment performed once with two cell lines n=3 images.

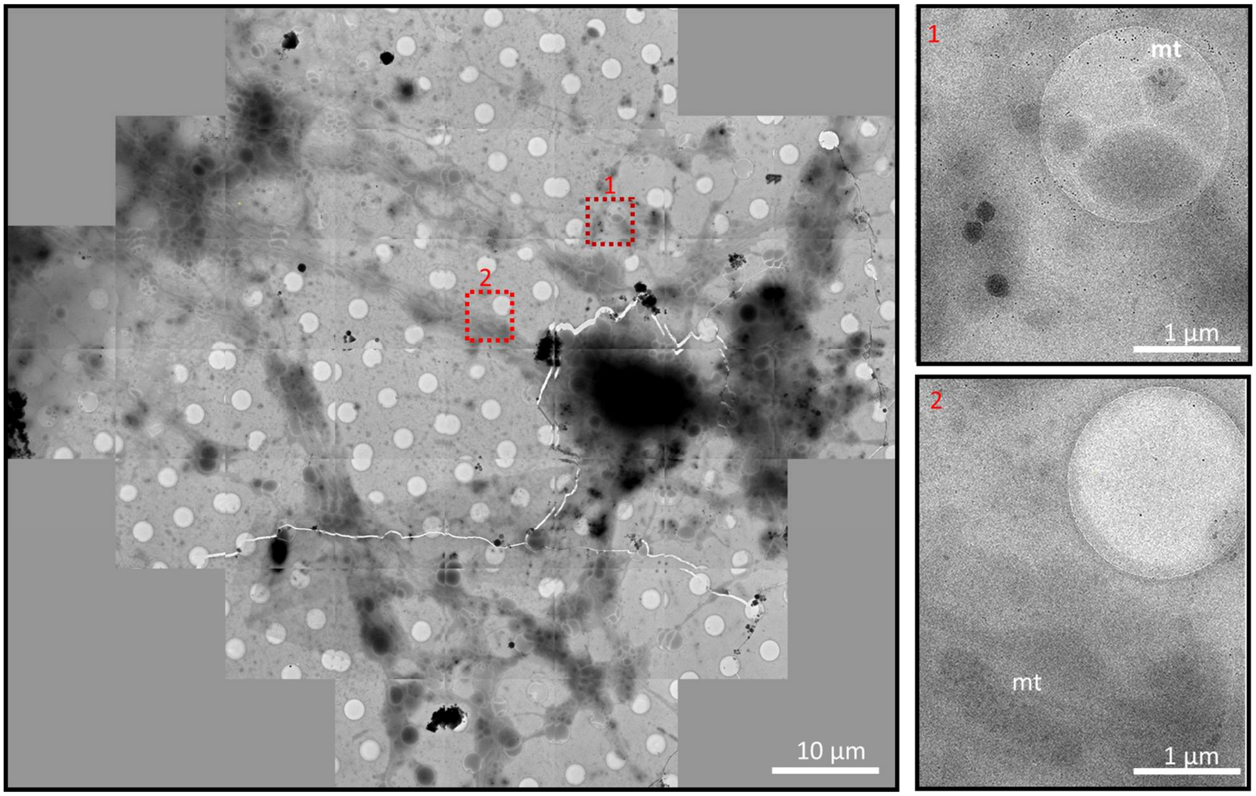

Supplementary Figure 2

Supplementary Fig. 2. HD patient iPSC-derived neurons grow well on cryoEM grids. A montage of low-magnification (6500 X) cryoEM images (n=56) of HD patient iPSC-derived neurons (left), and intermediate magnification (39000 X) screening images (right) from two regions highlighted with red boxes in the montage, showing putative mitochondria (labeled as mt) with visibly enlarged and dense granules inside. Scale bars = 10 **µ**m and 1 **µ**m.

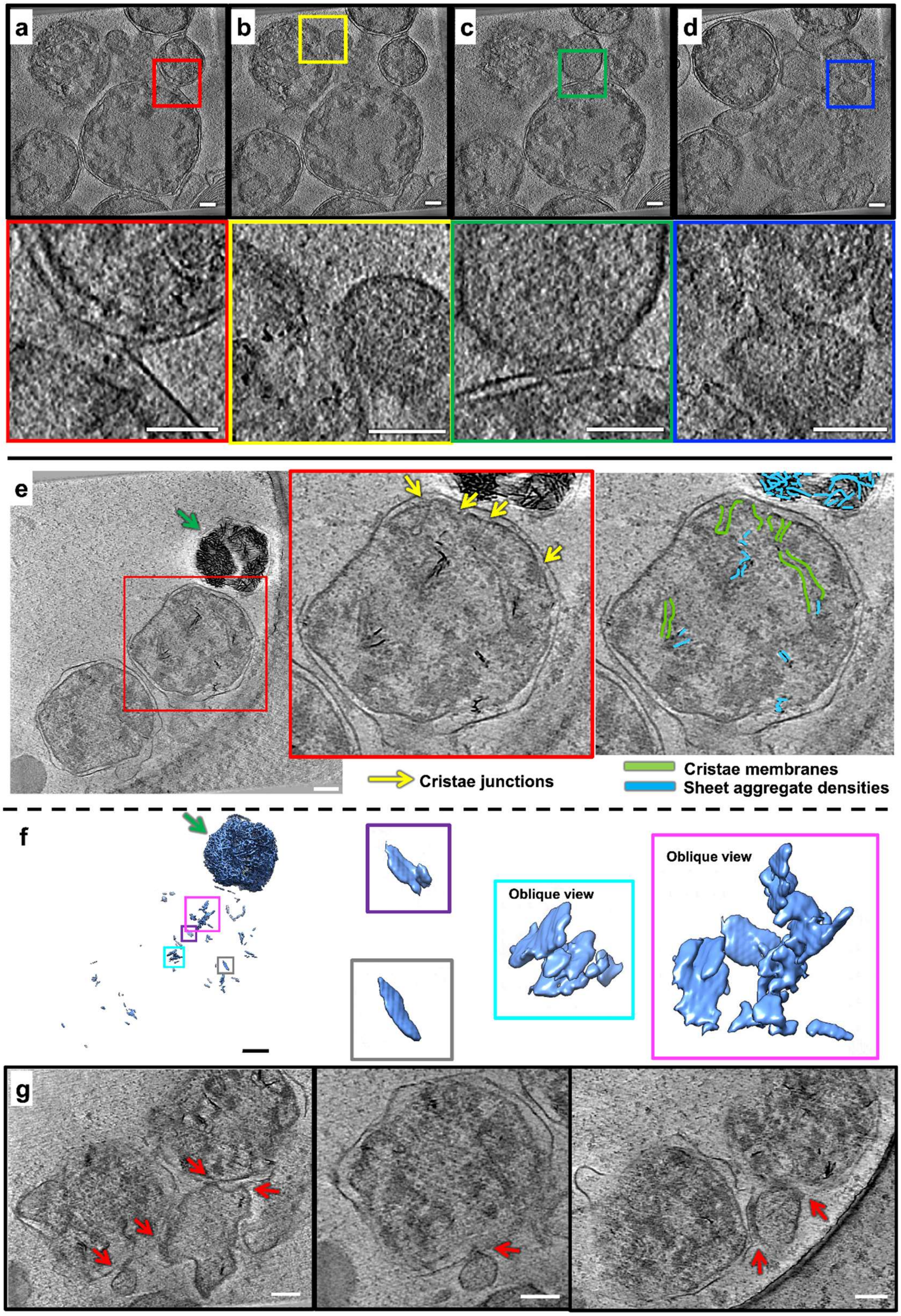

Supplementary Figure 3

Supplementary Fig. 3. Aggregates inside double membrane-bound compartments in a neurite of HD patient iPSC-derived neuron (Q53). Slices (~14 nm thick) through selected regions of a representative cryoET tomogram showing aggregates in double membrane-bound compartments (putatively organelles in the autophagic pathway) in a neurite of an HD patient iPSC-derived neuron (Q53), with blown-up views highlighting the compartments potentially fusing with **a** each other or **b-d** with single membrane-bound compartments (putatively lysosomes). **e** Slices (~1.4 nm thick) through another tomogram (Q53) showing 3 double membrane bound compartments, with the one in the top right of the image showing a compartment completely overwhelmed by sheet aggregates, and the one in the middle showing incipient sheet aggregate densities and structural hallmarks of mitochondria, such as a double membrane, cristae, and cristae junctions. **f** Semi-automated, neural-net based annotation with EMAN2 of sheet aggregate densities, training on a few positive references (n=10) from the sheet aggregate pointed at with the green arrow in the top right, identifies densities in the other membrane-bound compartments automatically as belonging to the same feature as the well-recognized, mature sheet aggregate in the top, free from bias. The blown-up and oblique (pink and cyan boxes) views on the right clearly show the sheet-like morphology of the incipient densities in the mitochondria-like compartment in the middle. **g** Tomographic slices (~56 nm thick) showing that the mitochondria-like organelle in e and its neighbor to the bottom-left are interacting with single membrane-bound compartments (at sites indicated by the red arrows), putatively lysosomes, like the double-membrane bound organelles shown in **a-g**. Scale bars are 100 nm.

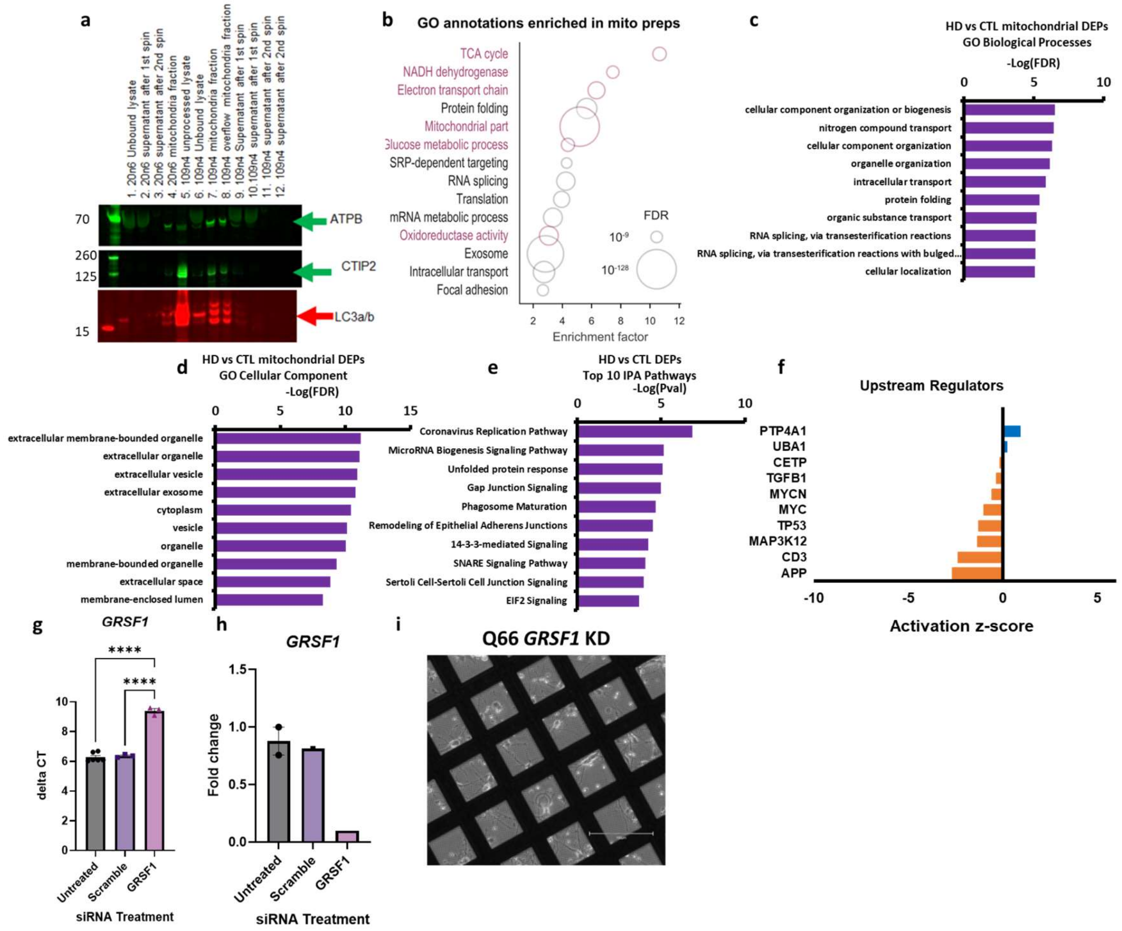

Supplementary Figure 4

Supplementary Fig. 4. Mass spectrometry of isolated mitochondria and *GRSF1* knockdown. **a** Representative Western blot on various fractions from the enrichment of mitochondria on a pilot experiment using MACS-based isolation probing for mitochondrial markers. **b** GO annotations show enrichment of mitochondrial proteins in isolated mitochondria from control and HD neurons. **c-f** IPA and GO analyses of HD vs control neurons highlighting significant DEPs that show the overrepresentation of proteins related to c GO biological processes and d GO cellular components. **e** Top 10 IPA pathways by p value and f Top 10 IPA Upstream regulators (genes and proteins) by p value that have assigned activation scores. Supplementary Data 1 shows full GO and IPA lists. **g-i** Q66 neurons were used for *GRSF1* knockdown with Accell siRNA at day 28 and cells harvested at day 37, **g** deltaCT (One-way ANOVA with Sidak's multiple comparison test F(2,9)=158.5 Scramble & GRSF1 n=3 and Untreated n=6 (technical replicates)) and **h** fold change (Scramble & GRSF1 n=1 and Untreated n=2) from RNA that was extracted for qPCR and showed significant knockdown compared to scramble negative control siRNA (padj<0.0001) and untreated neurons (padj<0.0001) **i** example of Q66 neurons on grids after *GRSF1* knockdown, data shows mean ± SEM. Source data is provided as a Source Data file.

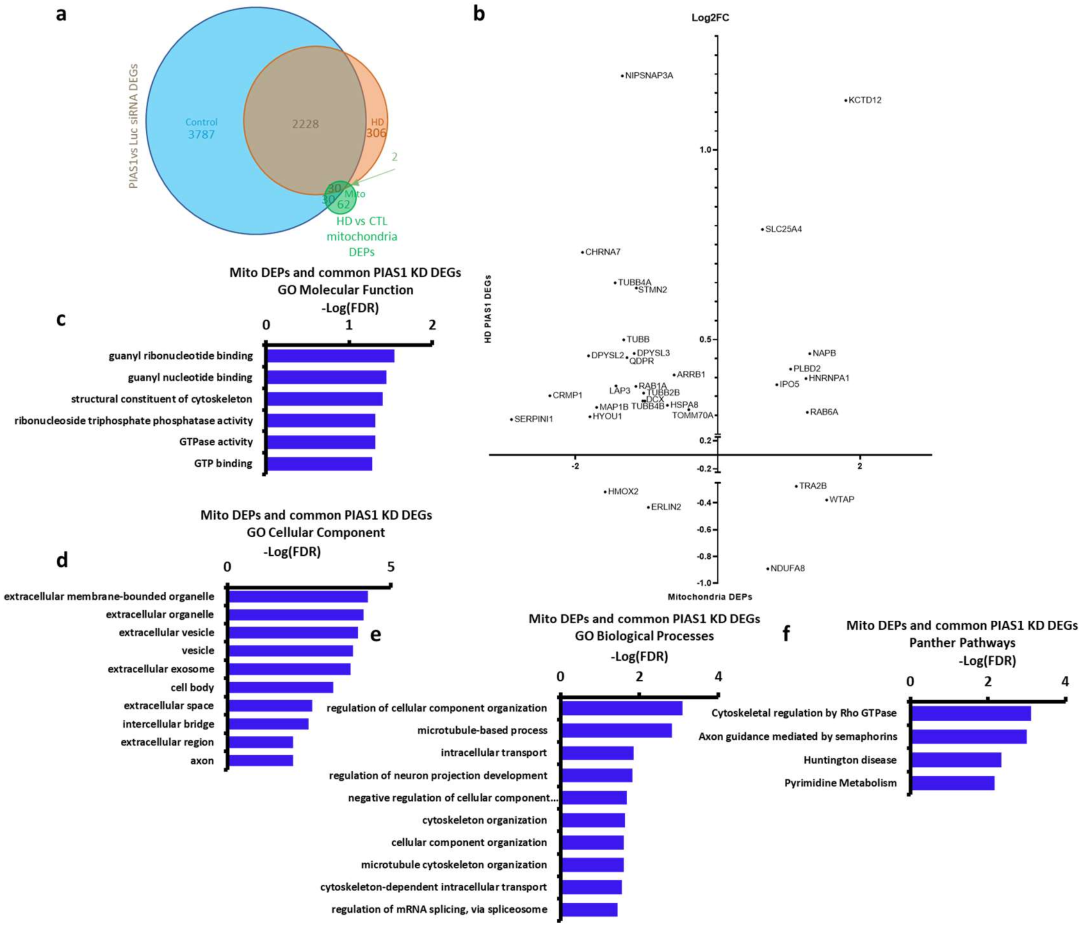

Supplementary Figure 5

Supplementary Fig. 5. Overlap of mitochondria DEPs and knockdown of PIAS1 DEGs. a Venn diagram that shows the total PIAS1 knockdown generated DEGs in control and HD iPSC-derived neurons and the overlap with the HD vs control mitochondrial DEPs. **b** Scatter plot of overlapping DEG log2 fold changes generated from PIAS1 knockdown in HD iPSC-derived neurons from previous work[2] plotted against the log2 fold enrichment of mitochondrial DEPs. **c-f** Assessing the 32 common genes between PIAS1 knockdown in HD neurons and HD mitochondria show overrepresentation of these terms for **c** Go Molecular function **d** GO Cellular component, **e** GO Biological processes and **f** Panther Pathways. **Supplementary Data 1** shows full GO terms lists.

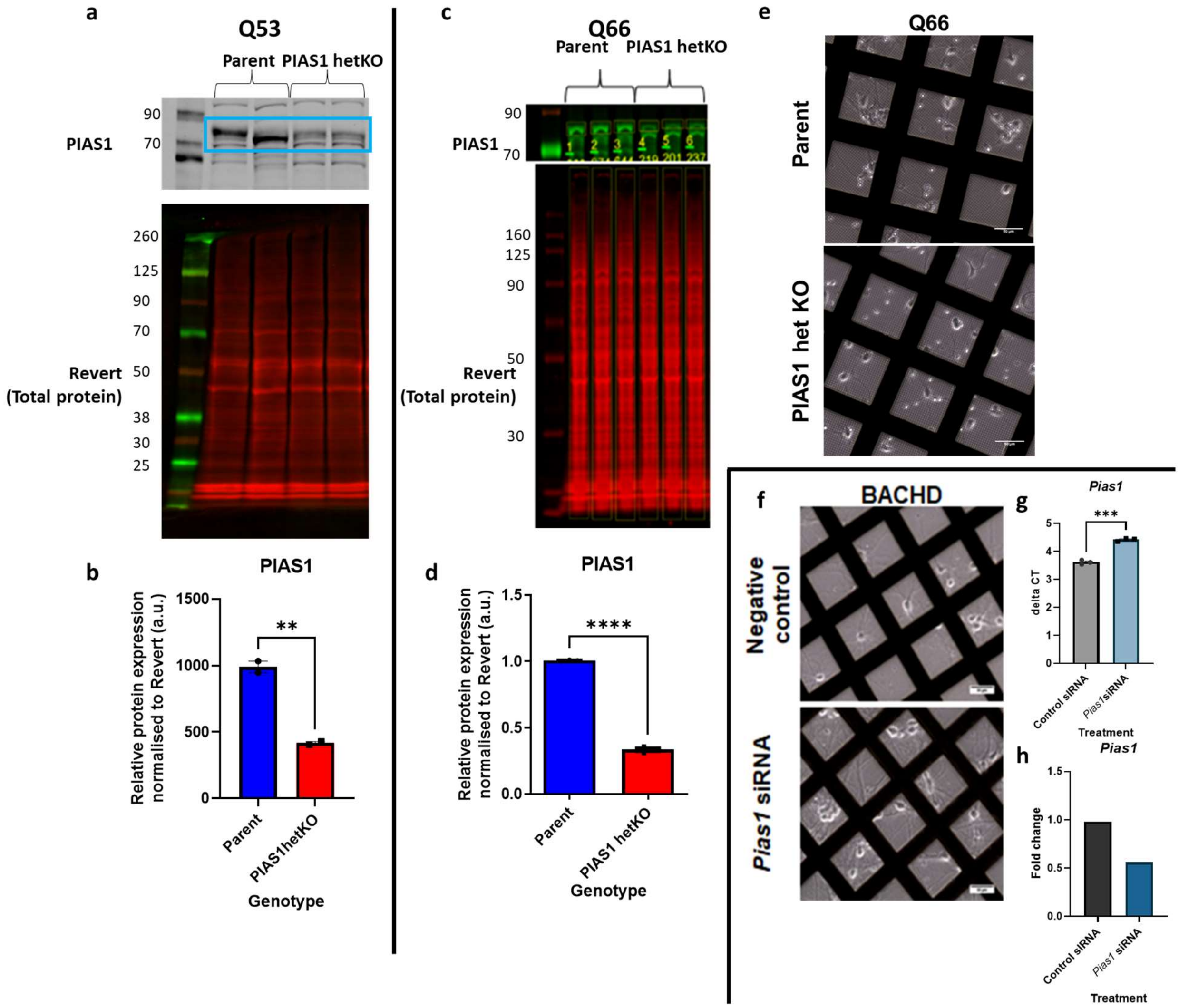

Supplementary Figure 6

Supplementary Fig. 6 Knockdown of PIAS1 a Western blot of day 16 neural progenitors of the 53Q iPSC line showing the parental and CRISPR edited line showing PIAS1 knockdown and this is quantified in **b** Unpaired two-tailed t-test t=12.93, df=2, p=0.0059 n=2 **c** Western blot of the 66Q iPSC line showing the parental and CRISPR edited line showing PIAS1 knockdown and this is quantified in **d** Unpaired two-tailed t-test t=46.70, df=4, p<0.0001 n=3. **e** Representative images of the Q66 iPSC derived neurons at day 37 prior to vitrification for cryoET showing PIAS1 knockdown does not affect cellular growth on the grids **f-h** E18 BACHD neurons at DIV14, Pias1 siRNA treatment was performed at DIV3, **f** Representative images of the primary neurons growing on grids prior to vitrification, **g-h** qRT-PCR for *Pias1* to validate *Pias1* knockdown in BACHD primary neurons, graphs show **g** delta CT (Unpaired two-tailed t-test t=14.96, df=4 p=0.0001) n=3 (technical replicates) and **h** Fold change showing significant knockdown n=1. Data shows mean ± SEM. Source data is provided as a Source Data file.

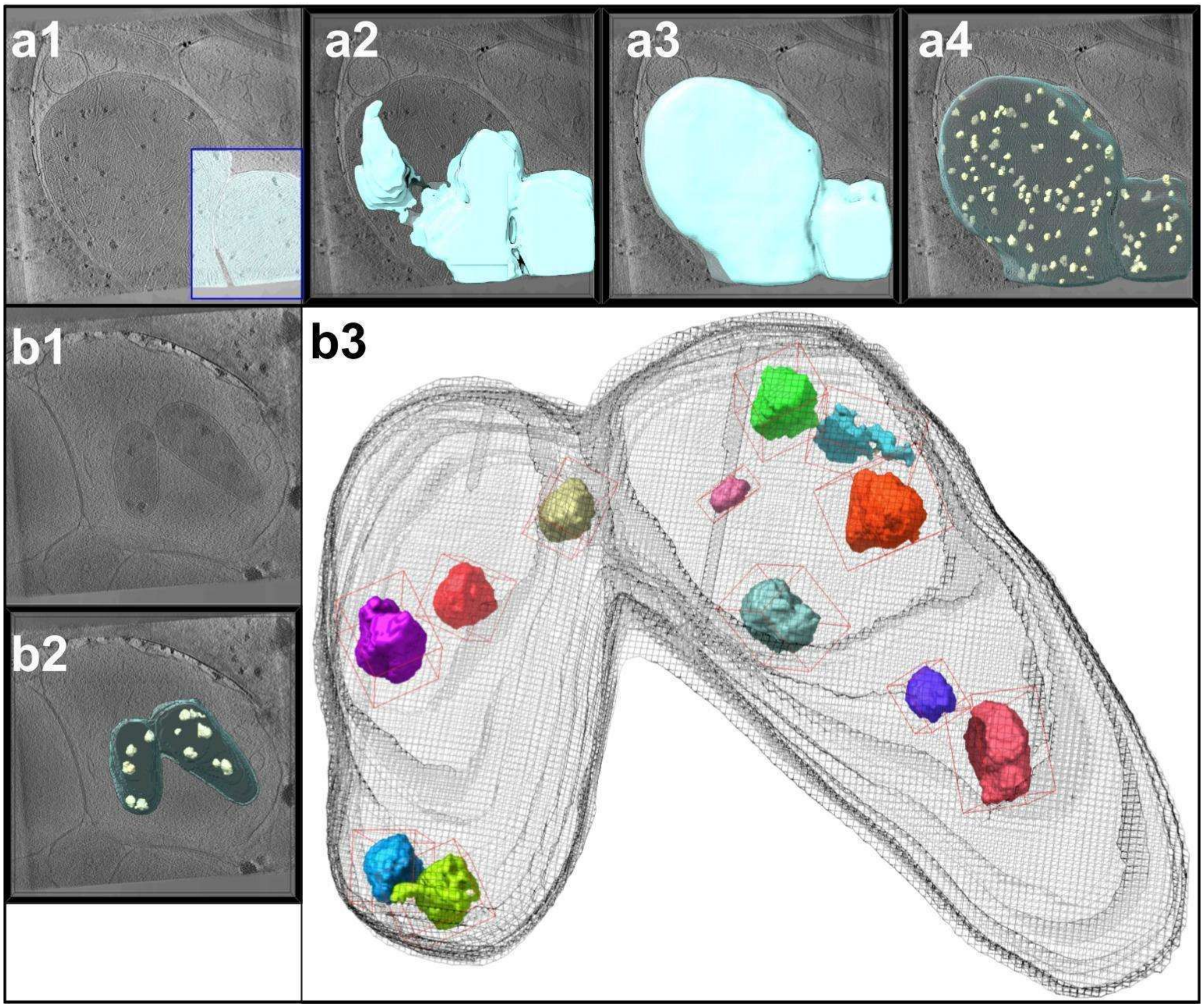

Supplementary Figure 7

Supplementary Fig. 7. Granule and Mitochondria Segmentation Pipeline. a1 - The 3D UNet for mitochondrial segmentation is trained on a handful of partially annotated slices. Pixels in the blue rectangle are labeled as being part of the mitochondria or the background and the rest are unlabelled. **a2** - High confidence predictions (shown in cyan) from the 3D UNet are used as pseudo-labels to augment the training set. A new 3D UNet is trained on the augmented dataset. **a3** - Retraining the 3D UNet on this augmented dataset improves segmentation quality. The new segmentation (shown in cyan) spans the full extent of the mitochondria. **a4** - The 3D UNet used to detect granules is applied to the mitochondrial volume. The resulting predictions are shown in yellow. **b1** - A previously unseen tomogram is fed into the trained 3D UNets. **b2** - The mitochondria and granule predictions produced by the segmentation pipeline. **b3** - A connected components analysis is used to identify individual granules and measure their volumes.

Supplementary Fig 4a

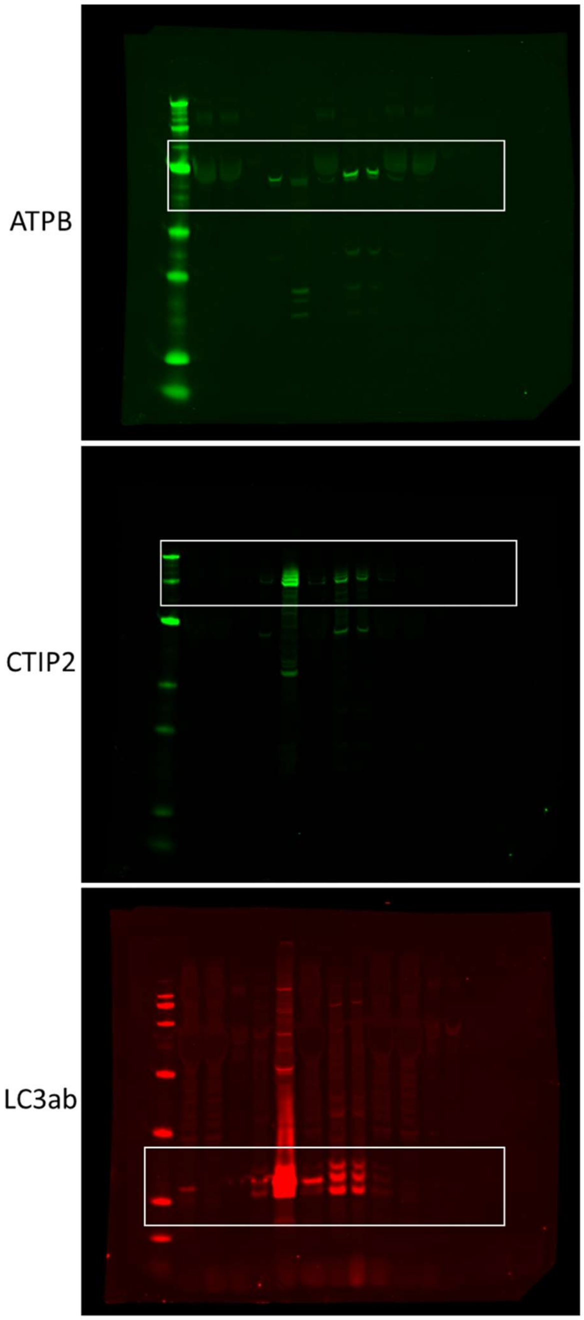

Supplementary Fig 6a and 6c

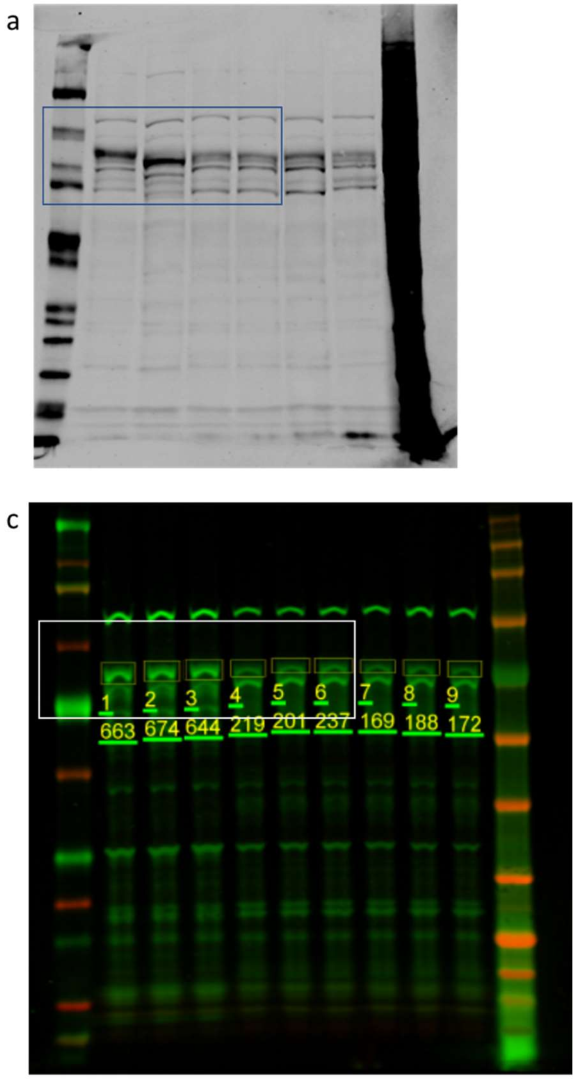

Supplementary Figure 8

Supplementary Fig. 8. Complete Gels from Images shown in Supplementary Figs. 4 and 6. - The boxes indicate the portions shown in **Supplementary Figs. 4a (top), 6a (top) and 6c (top).**

Supplementary Table 1. Number of EM grids in iPSC-differentiation or mouse primary neuron petri dishes and tilt series collected from them, reconstructed into tomograms. "M" in the second to last column indicates the number of tomograms containing mitochondria with visible granules used for quantification in **Fig. 9** while "A" indicates the number of tomograms containing large sheet aggregates.

**iPSC**	**Q18**	No. of EM grids	3
		No. of final tomograms	46
		No. of mitochondria	21
		No. of sheet aggregates	0
	**Q20**	No. of EM grids	3
		No. of final tomograms	50
		No. of mitochondria	20
		No. of sheet aggregates	0
	**Q53**	No. of EM grids	3
		No. of final tomograms	46
		No. of mitochondria	14
		No. of sheet aggregates	5
	**Q53 *PIAS1* hetKO**	No. of EM grids	3
		No. of final tomograms	23
		No. of mitochondria	22
		No. of sheet aggregates	0
	**Q66**	No. of EM grids	3
		No. of final tomograms	31
		No. of mitochondria	10
		No. of sheet aggregates	8
	**Q66 *PIAS1* hetKO**	No. of EM grids	3
		No. of final tomograms	71
		No. of mitochondria	63
		No. of sheet aggregates	3
	**Q66 *GRSF1* KD**	No. of EM grids	3
		No. of final tomograms	17
		No. of mitochondria	17
		No. of sheet aggregates	0
	**Q77**	No. of EM grids	3
		No. of final tomograms	10
		No. of mitochondria	5
		No. of sheet aggregates	6
	**Q109**	No. of EM grids	3
		No. of final tomograms	42

		No. of mitochondria	33
		No. of sheet aggregates	6
Mouse	WT	No. of EM grids	3
		No. of final tomograms	34
		No. of mitochondria	31
		No. of sheet aggregates	0
	BACHD	No.of EM grids	3
		No. of final tomograms	24
		No. of mitochondria	22
		No. of sheet aggregates	14
	BACHD RNAi control	No. of EM grids	3
		No. of final tomograms	29
		No. of mitochondria	5
		No. of sheet aggregates	3
	BACHD *Pias1* KD	No. of EM grids	3
		No. of final tomograms	36
		No. of mitochondria	12
		No. of sheet aggregates	5
	dN17-BACHD	No. of EM grids	3
		No. of final tomograms	34
		No. of mitochondria	15
		No. of sheet aggregates	6

Supplementary Table 2: Kruskal-Wallis statistics that pertain to data displayed in **Fig. 9**

**Sample**	**Measurement**	**Kruskal-Wallis summary**	**Multiple comparison test**	**Multiple comparisons stats**
Human iPSC-neurons	Granule volume	K-W Stat = 857.2 No. of groups = 9, P value <0.0001, no. of values = 6764	Dunn's	Q18 vs. Q20 padj=0.7569
				Q18 vs. Q53 padj<0.0001
				Q18 vs. Q66 padj<0.0001
				Q18 vs. Q77 padj<0.0001
				Q18 vs. Q109 padj=0.0603
				Q53 vs. Q109 padj<0.0001
				Q20 vs. Q53 padj<0.0001
				Q20 vs. Q66 padj<0.0001
				Q20 vs. Q77 padj<0.0001
				Q20 vs. Q109 padj>0.9999
				Q66 vs. Q66 PIAS1 hetKO padj<0.0001
				Q20 vs. Q53 PIAS1 hetKO padj<0.0001
				Q18 vs. Q53 PIAS1 hetKO padj=0.0002
				Q53 vs. Q53 PIAS1 hetKO padj<0.0001
				Q66 vs. Q66 *GRFS1* siRNA padj<0.0001
				Q18 vs. Q66 *GRFS1* siRNA padj<0.0001
				Q20 vs. Q66 *GRFS1* siRNA padj<0.0001
				Q18 vs. Q66 PIAS1 hetKO padj=0.1704

				Q20 vs. Q66 PIAS1 hetKO padj>0.9999
Mouse primary neurons	Granule volume	K-W Stat = 1030 No. of groups = 5 P value<0.0001 , no. of values = 3351	Dunn's	WT vs. BACHD padj<0.0001
				WT vs. dN17-BACHD padj<0.0001
				WT vs. BACHD Control siRNA padj<0.0001
				WT vs. BACHD *Pias1* siRNA padj>0.9999
				BACHD Control siRNA vs. BACHD *Pias1* siRNA padj<0.0001
				BACHD vs. BACHD Control siRNA padj=0.0119
				BACHD vs. BACHD *Pias1* siRNA padj<0.0001
				BACHD vs. dN17-BACHD padj<0.0001
Human iPSC-neurons	Granule count/nm^3 of mitochondria	K-W Stat = 129.6 No. of groups = 9, P value<0.0001 , no. of values = 302	Dunn's	Q18 vs. Q20 padj>0.9999
				Q18 vs. Q53 padj=0.0230
				Q18 vs. Q66 padj>0.9999
				Q18 vs. Q77 padj=0.4068
				Q18 vs. Q109 padj>0.9999
				Q53 vs. Q109 padj=0.7210
				Q20 vs. Q53 padj=0.0104
				Q20 vs. Q66 padj>0.9999
				Q20 vs. Q77 padj=0.3234
				Q20 vs. Q109 padj=0.9524
				Q18 vs. Q53 PIAS1 hetKO padj<0.0001
				Q20 vs. Q53 PIAS1 hetKO padj<0.0001

				Q53 vs. Q53 PIAS1 hetKO padj>0.9999
				Q18 vs. Q66 *GRFS1* siRNA padj=0.0104
				Q20 vs. Q66 *GRFS1* siRNA padj=0.0040
				Q66 vs. Q66 *GRFS1* siRNA padj=0.0316
				Q18 vs. Q66 PIAS1 hetKO padj=0.0994
				Q20 vs. Q66 PIAS1 hetKO padj=0.0402
				Q66 vs. Q66 PIAS1 hetKO padj=0.2373
Mouse primary neurons	Granule count/nm^3 of mitochondria	K-W Stat = 68.28 No. of groups = 5 P value<0.0001, no. of values = 104	Dunn's	WT vs. BACHD padj<0.0001
				WT vs. dN17-BACHD padj<0.0001
				WT vs. BACHD Control siRNA padj<0.0001
				WT vs. BACHD *Pias1* siRNA padj<0.0001
				BACHD Control siRNA vs. BACHD *Pias1* siRNA padj>0.9999
				BACHD vs. BACHD Control siRNA padj>0.9999
				BACHD vs. BACHD *Pias1* siRNA padj>0.9999
				BACHD vs. dN17-BACHD padj>0.9999

Supplementary Table 3: iPSC line information

Q length	Cell line	Fibroblast number	Sex	Reprogramming method	Clinical notes	Publications
**18Q**	CS25iCTR18n6	ND30625	XY	Non-integrating episomal	Normal, brother of affected sibling	[1]
**20Q**	CS71iCTR20n6	ND29971	XX	Non-integrating episomal	Normal	[2]
**53Q**	CS03iHD53n3	UCI-HDF3	XY	Non-integrating episomal	Clinically affected	[1]
**66Q**	CS02iHD66n4	UCI-HDF2	XX	Non-integrating episomal	Clinically affected, family history unknown. Age of onset 16 years	[1,2]
**77Q**	CS77iHD77n3	JHU77	XY	Non-integrating episomal	Clinically affected	[3]
**109Q**	CS09iHD109n1	JHU109	XX	Non-integrating episomal	Clinically Affected; Juvenile onset form with severe bradykinesia, rigidity and dystonia at time of	[1]

					biopsy. Age of onset: 3 years	

Supplementary References

1. Smith-Geater, C. *et al.* Aberrant Development Corrected in Adult-Onset Huntington's Disease iPSC-Derived Neuronal Cultures via WNT Signaling Modulation. *Stem Cell Reports* **14**, 406–419 (2020).
2. Morozko, E. L. *et al.* PIAS1 modulates striatal transcription, DNA damage repair, and SUMOylation with relevance to Huntington's disease. *Proc. Natl. Acad. Sci. U. S. A.* **118**, (2021).
3. Cedars-Sinai Biomanufacturing Center; https://biomanufacturing.cedars-sinai.org/product/cs77ihd-77nxx/
