# Supplementary material for: CryoET reveals organelle phenotypes in huntington disease patient iPSC-derived and mouse primary neurons
Source: Nat Commun. 2023 Feb 8;14:692. doi: 10.1038/s41467-023-36096-w (PMC9908936; doi:10.1038/s41467-023-36096-w)
Supplement: Supplementary file 3 — Description of Additional Supplementary Files [file 41467_2023_36096_MOESM3_ESM.pdf]

File name: Supplementary Data 1

Description: Mitochondria DEPs, downstream analysis and PIAS1 DEGs from previous study [2]. Refer to .XLS File

File name: Supplementary Movie 1

Description: Representative cryoET tomogram of a neurite in an HD patient iPSC-derived neuron (Q77) containing a prominent mitochondrion with aberrantly enlarged granules in the mitochondrial matrix composed of tightly packed, heterogeneous densities. Segmentation colors: red: microtubules, yellow: mitochondrial double-membranes, dark blue: granules, and cyan: cristae membranes.

File name: Supplementary Movie 2

Description: Representative cryoET tomogram of a neurite in an HD patient iPSC-derived neuron (Q66) displaying a double membrane-bound compartment with a large sheet aggregate. Segmentation colors: orange: sheet aggregate, cerulean blue: double membrane.
