# Supplementary material for: CryoET reveals organelle phenotypes in huntington disease patient iPSC-derived and mouse primary neurons
Source: Nat Commun. 2023 Feb 8;14:692. doi: 10.1038/s41467-023-36096-w (PMC9908936; doi:10.1038/s41467-023-36096-w)
Supplement: Supplementary file 7 — Reporting Summary [file 41467_2023_36096_MOESM7_ESM.pdf]

nature portfolio

Corresponding author(s): Wah Chiu & Leslie M. Thompson

Last updated by author(s): Dec 16, 2022

Reporting Summary

Nature Portfolio wishes to improve the reproducibility of the work that we publish. This form provides structure for consistency and transparency in reporting. For further information on Nature Portfolio policies, see our Editorial Policies and the Editorial Policy Checklist.

Statistics

For all statistical analyses, confirm that the following items are present in the figure legend, table legend, main text, or Methods section.

n/a	Confirmed	
☐	☒	The exact sample size (*n*) for each experimental group/condition, given as a discrete number and unit of measurement
☐	☒	A statement on whether measurements were taken from distinct samples or whether the same sample was measured repeatedly
☐	☒	The statistical test(s) used AND whether they are one- or two-sided<br>*Only common tests should be described solely by name; describe more complex techniques in the Methods section.*
☐	☒	A description of all covariates tested
☐	☒	A description of any assumptions or corrections, such as tests of normality and adjustment for multiple comparisons
☐	☒	A full description of the statistical parameters including central tendency (e.g. means) or other basic estimates (e.g. regression coefficient) AND variation (e.g. standard deviation) or associated estimates of uncertainty (e.g. confidence intervals)
☐	☒	For null hypothesis testing, the test statistic (e.g. *F*, *t*, *r*) with confidence intervals, effect sizes, degrees of freedom and *P* value noted<br>*Give P values as exact values whenever suitable.*
☒	☐	For Bayesian analysis, information on the choice of priors and Markov chain Monte Carlo settings
☒	☐	For hierarchical and complex designs, identification of the appropriate level for tests and full reporting of outcomes
☒	☐	Estimates of effect sizes (e.g. Cohen's *d*, Pearson's *r*), indicating how they were calculated

Our web collection on statistics for biologists contains articles on many of the points above.

Software and code

Policy information about availability of computer code

Data collection	SerialEM version 3.90 beta
Data analysis	IMOD version 4.12.9, EMAN2 version 2.99, UCSF Chimera version Alpha 1.17. The software and AI models we developed to segment the mitochondria and granules will be uploaded to GitHub upon publication. Prism was used for statistical analysis version 9.3 Raw MS data were processed using MaxQuant version 1.6.7.0, Statistical tests for Mass spec were performed with Perseus version 1.6.7.0 using either ProteinGroups or Peptides output tables from MaxQuant. MS/MS spectra searches were performed using the Andromeda search engine(Cox et al. 2011) against the forward and reverse human and mouse Uniprot databases (downloaded August 28, 2017 and November 25, 2020, respectively). QIAGEN Ingenuity Pathway Analysis version 81348237 was used for further analysis of MS data. Gene ontology analysis was done using dataset on http://www.pantherdb.org using GO Ontology database DOI: 10.5281/zenodo.6799722 Released 2022-07-01 and PANTHER version 17.0 Released 2022-02-22. The software and AI models we developed to segment the mitochondria and granules are accessible at GitHub (https://github.com/sanketx/mitochondria_segmentation).

For manuscripts utilizing custom algorithms or software that are central to the research but not yet described in published literature, software must be made available to editors and reviewers. We strongly encourage code deposition in a community repository (e.g. GitHub). See the Nature Portfolio guidelines for submitting code & software for further information.

Data

Policy information about availability of data

All manuscripts must include a data availability statement. This statement should provide the following information, where applicable:

- Accession codes, unique identifiers, or web links for publicly available datasets
- A description of any restrictions on data availability
- For clinical datasets or third party data, please ensure that the statement adheres to our policy

We will deposit representative tomograms for each phenotype in EMDB (https://www.emdataresource.org/deposit.html);
Representative tomograms for each phenotype have been deposited in the EMDB (https://www.emdataresource.org/deposit.html) with accession codes as follows:
EMD-28668 : iPSC Q18 neuron with enlarged mitochondrial granules.
EMD-28944 : iPSC Q20 neuron with enlarged mitochondrial granules.
EMD-28946 : iPSC Q53 neuron with enlarged mitochondrial granules.
EMD-29074 : iPSC Q66 neuron with enlarged mitochondrial granules.
EMD-29075 : iPSC Q77 neuron with enlarged mitochondrial granules.
EMD-29076 : iPSC Q109 neuron with enlarged mitochondrial granules.
EMD-29080 : iPSC Q66 neuron with PIAS1-hetKO treatment showing normal mitochondrial granules.
EMD-29081 : iPSC Q66 with GRSF1-KD treatment showing normal mitochondrial granules.
EMD-29210 : mitochondria purified from iPSC Q109 neuron with enlarged mitochondrial granules.
EMD-29083 : WT mouse neuron showing normal mitochondria.
EMD-29207 : BACHD mouse model neuron with enlarged mitochondrial granules.
EMD-29084 : BACHD-dN17 mouse model neuron with enlarged mitochondrial granules.
EMD-29079 : iPSC Q66 neuron with sheet aggregates in autophagic organelles.
EMD-29211 : iPSC Q66 neuron with PIAS1-hetKO treatment rescues sheet aggregates in autophagic organelles.
EMD-29208 : BACHD mouse model neuron with sheet aggregates in autophagic organelles.

The mass spectrometry proteomics data have been deposited to the ProteomeXchange Consortium via the PRIDE partner repository with the dataset identifier PXD037526. PIAS1 knockdown RNAseq dataset that was previously published (Morozko et al., 2021) GEO (GSE162349) was used for comparison to the mitochondria mass spec reported here. MS/MS spectra searches were performed against the forward and reverse human and mouse Uniprot databases (downloaded August 28, 2017 and November 25, 2020, respectively).

Human research participants

Policy information about studies involving human research participants and Sex and Gender in Research.

Reporting on sex and gender	*Use the terms sex (biological attribute) and gender (shaped by social and cultural circumstances) carefully in order to avoid confusing both terms. Indicate if findings apply to only one sex or gender; describe whether sex and gender were considered in study design whether sex and/or gender was determined based on self-reporting or assigned and methods used. Provide in the source data disaggregated sex and gender data where this information has been collected, and consent has been obtained for sharing of individual-level data; provide overall numbers in this Reporting Summary. Please state if this information has not been collected. Report sex- and gender-based analyses where performed, justify reasons for lack of sex- and gender-based analysis.*
Population characteristics	*Describe the covariate-relevant population characteristics of the human research participants (e.g. age, genotypic information, past and current diagnosis and treatment categories). If you filled out the behavioural & social sciences study design questions and have nothing to add here, write "See above."*
Recruitment	*Describe how participants were recruited. Outline any potential self-selection bias or other biases that may be present and how these are likely to impact results.*
Ethics oversight	*Identify the organization(s) that approved the study protocol.*

Note that full information on the approval of the study protocol must also be provided in the manuscript.

Field-specific reporting

Please select the one below that is the best fit for your research. If you are not sure, read the appropriate sections before making your selection.

☒ Life sciences ☐ Behavioural & social sciences ☐ Ecological, evolutionary & environmental sciences

For a reference copy of the document with all sections, see nature.com/documents/nr-reporting-summary-flat.pdf

Life sciences study design

All studies must disclose on these points even when the disclosure is negative.

Sample size	For cryoET, we describe sample sizes for all human iPSC and mouse model cell lines through the text, and summarize in Supp. Table 1 , for mass spec we have previously used n=3 for sample preparation which was sufficient to provide statistically significant differences. For quantifying PIAS1 knockdown we used 2 (53Q) or 3 (66Q) iPSC wells to determine the knockdown and 2 samples (one NT - not shown and one HD) sample in technical triplicate for the mouse primary neuron samples due to limited number of cells to create cryoET samples. For immunofluorescence QC one coverslip per cell line (2 cell lines total) was used to determine the efficiency of DARPP32/CTIP2 neuron generation.
Data exclusions	For cryoET, we eliminated individual "bad" images from some tilt series if they showed artifacts such as incorrectible drift or grid bars
Replication	For cryoET, for each specimen, tomograms were collected from 3 individual EM grids, cells were grown in multiple batches (at least 5) for data collection and observed throughout the duration of data generation however not all used for the data in this manuscript. For mitochondria MS analysis 3 replicates per one cell line were provided, this experiment was performed once to try and ensure variability is produced by HD vs control rather than batch to batch variability of differentiations.
Randomization	For all specimens, mitochondria and double-membrane organelles in neurites were randomly selected for cryoET imaging. For imaging of immunofluorescence random fields of view were chosen based on available nuclei in field of view<br><br>Comment from editors: For all specimens please clearly describe how samples were allocated into experimental groups. If allocation was not random, describe how covariates were controlled OR if this is not relevant to your study, explain why.
Blinding	For cryoET, each condition required a sufficient number of grids and tomograms to evaluate so the user was not blinded. For AI analysis, the individual was blinded to condition. For immunofluorescence blinding was not performed as we were not quantifying results and was used for QC rather than data generation. For Mass spec, the user was not blinded as samples were processed and analyzed in parallel to reduce potential technical variability, and knowledge of the sample identity is required for any downstream statistical analyses

Reporting for specific materials, systems and methods

We require information from authors about some types of materials, experimental systems and methods used in many studies. Here, indicate whether each material, system or method listed is relevant to your study. If you are not sure if a list item applies to your research, read the appropriate section before selecting a response.

Materials & experimental systems

n/a	Involved in the study	
☐	☒	Antibodies
☐	☒	Eukaryotic cell lines
☒	☐	Palaeontology and archaeology
☐	☒	Animals and other organisms
☒	☐	Clinical data
☒	☐	Dual use research of concern

Methods

n/a	Involved in the study	
☒	☐	ChIP-seq
☒	☐	Flow cytometry
☒	☐	MRI-based neuroimaging

Antibodies

Antibodies used	LC3ab (Cell signaling 12741 lot#5), PIASl (Cell Signaling 3550 lot#3), PIASl (Proteintech 23395-1-AP lot#00054319), DARPP32 (Abcam ab40801 lot#GR3362694-4), CTIP2 (Abcam ab18465, lot#GR3272266-11), ATPB (Abcam ab14730 lot#GR3301224-9)
Validation	For immunofluorescence, staining was performed alongside species appropriate IgG isotypes and exposure for staining set to that limit. For Western blot, only size appropriate bands were considered. For the PIASl antibodies, this has been validated in our lab using knockdown experiments (Morozko et al., 2021) and in an additional publication for PIAS1 Proteintech antibody (DOI: 10.1371/journal.ppat.1010446) and our previously validated knockdown lines. For CTIP2 knockout validation has been performed using IHC (https://www.jstage.jst.go.jp/article/expanim/60/4/60_4_355/_pdf/-char/en) but not western. For LC3a/b validation by western for knockout was performed using HEK293 cells in previously published article (https://www.ncbi.nlm.nih.gov/pmc/articles/PMC6419514/). For DARPP32 and ATPB knockout validation has not yet been performed,

Eukaryotic cell lines

Policy information about cell lines and Sex and Gender in Research

Cell line source(s)	Generation and characterization of iPSC lines: The control lines (termed 18Q and 20Q) and 4 HD iPSC lines (termed 53Q, 66Q, 77Q and 109Q) were generated using episomal plasmids and characterized as previously described (Sareen et al., 2013). CRISPR editing of the 66Q line was previously described (Morozko et al., 2021). Fibroblasts from control (18Q, 20Q) and from

	HD patients (53Q, 66Q, 77Q and 109Q) were obtained from several sources 18Q - Coriell Fibroblast ND30625, 20Q - Coriell Fibroblast ND29971, 53Q - University of California Irvine (UCI) HDF3, 66Q - UCI HDF2, 77Q - Johns Hopkins University (JHU) JHU77 and 109Q - JHU109. The Coriell Cell Repository maintains the consent and privacy of the donor of fibroblast samples. All the cell lines and protocols in the present study were carried out in accordance with the guidelines approved by institutional review boards at the UCI and JHU. Studies were performed under the auspices of the UCI Institutional Review Board (IRB) approved protocol UCI IRB #2008-6556. The reprogramming and characterization of iPSC lines was performed at Cedars Sinai in accordance with the guidelines approved by Stem Cell Research Oversight (SCRO) committee and IRB under the auspices IRB-SCRO Protocol Pro00032834 (iPSC Core Repository and Stem Cell Program). Maintenance and differentiation of iPSCs were performed at UCI in accordance with the protocol at UCI hSCRO #2008-6556. Appropriate informed consents were obtained from all the donors. All of the iPSC lines described in this study are available from the iPSC Core at the Cedars-Sinai Biomanufacturing Center iPSC repository. To protect donor privacy and confidentiality, all samples were coded and de-identified in this study. To assess purity and confirm donor identity of parental tissue (fibroblasts), reprogrammed iPSCs and differentiated neurons, quality control was implemented assessing differentiation capacity to neurons by morphology and by neuron and MSN marker staining. G-band karyotyping/aCgH array was performed to ensure that iPSCs maintained normal karyotypes (46XX or 46XY where appropriate).
Authentication	Cells were defined as pluripotent prior to differentiation by staining and morphology,
Mycoplasma contamination	Cells were tested for mycoplasma, and were all negative
Commonly misidentified lines (See ICLAC register)	*Name any commonly misidentified cell lines used in the study and provide a rationale for their use.*

Animals and other research organisms

Policy information about studies involving animals; ARRIVE guidelines recommended for reporting animal research, and Sex and Gender in Research

Laboratory animals	The background for WT, BACHD and deltaN17 BACHD neurons is FVB/N-Tg(HTT*97Q)IXwy/J. E18 embryos of both male and female were used in the study. Animals are housed in a 12/12 light/dark cycle with ambient humidity (30-70%) and temperature controlled (68-72ºF)
Wild animals	No wild animals used
Reporting on sex	As pups were pooled for primary neuron generation sex ratios were unknown
Field-collected samples	No field collected samples were used in the study
Ethics oversight	IACUC approval from University of California in San Diego was in place for laboratory animal work

Note that full information on the approval of the study protocol must also be provided in the manuscript.
